# Supplementary material for: Large anomalies in future extreme precipitation sensitivity driven by atmospheric dynamics
Source: Nat Commun. 2023 Jun 2;14:3197. doi: 10.1038/s41467-023-39039-7 (PMC10238374; doi:10.1038/s41467-023-39039-7)
Supplement: Supplementary file 1 — Supplementary Information [file 41467_2023_39039_MOESM1_ESM.pdf]

Supplementary Information for

Large anomalies in future extreme precipitation sensitivity driven by atmospheric dynamics

Lei Gu[1,2], Jiabo Yin[1*], Pierre Gentine[3,4], Hui-Min Wang[5], Louise J. Slater[6], Sylvia C. Sullivan[7], Jie Chen[1], Jakob Zscheischler[8], Shenglian Guo[1]

[1]State Key Laboratory of Water Resources and Hydropower Engineering Science, Wuhan University, Wuhan 430072, China

[2]Hubei Key Laboratory of Digital River Basin Science and Technology, Huazhong University of Science and Technology, Wuhan 430074, China

[3]Department of Earth and Environmental Engineering, Columbia University, New York, NY, USA

[4]Climate School, Columbia University, New York, NY 10025, USA

[5]Department of Civil and Environmental Engineering, National University of Singapore, Singapore

[6]School of Geography and the Environment, University of Oxford, Oxford, UK

[7] Department of Chemical & Environmental Engineering, University of Arizona, Tucson, Arizona

[8]Department of Computational Hydrosystems, Helmholtz Centre for Environmental Research, Leipzig, Germany

*Corresponding author. Email: jboyn@whu.edu.cn;

Contents of this Supplementary Information:

Summary about supplementary information

Supplementary Figures 1 to 17

Supplementary Tables 2

Summary about supplementary information

This supplementary file includes 17 figures and 2 tables:

Supplementary Fig. 1 evaluates the robustness of the physical diagnostic approach in estimating extreme precipitation sensitivity (EPS) during the historical and future periods.

Supplementary Fig. 2 verifies the robustness of the binning scaling approach by using the quantile regression and the Akaike information criterion.

Supplementary Figs. 3-5 serve for the “Decomposition of thermodynamic and dynamic contributions” section. We demonstrate the thermodynamic versus dynamic scaling (Supplementary Fig. 2) and show the decomposed three thermodynamic (i.e., *pPR*, *pT* and *pLR*) and one dynamic (i.e., *DY*) scaling rates (Supplementary Fig. 3) as well as associated T_{pp} (Supplementary Fig. 4) during the historical period.

Supplementary Figs. 6-11 serve for the “Shifting thermodynamic and dynamic controls under climate change” section. We firstly analyze the T_{pp} changes (Supplementary Fig. 5) and local mean temperature (T_m) changes (Supplementary Fig. 6) between the 1985-2014 reference and 2071-2100 future periods. We then compare differences between T_{pp} and T_m during the reference (Supplementary Fig. 7) and future (Supplementary Fig. 8) periods, respectively, to fully investigate local warming impacts on the shifting EPS. Finally, we decompose the EPS and T_{pp} changes between 1985-2014 reference and 2071-2100 future periods into three thermodynamic and one dynamic components (Supplementary Figs. 9-10) to show how each term contributes to the total changes.

Supplementary Figs. 12-13 explore the synoptic patterns behind extreme precipitation. We use four indices (including CAPE, CIN, TCWV and VIMD) to analyze the physics behind extreme precipitation. We estimate their anomalies during extreme precipitation (Supplementary Fig. 12) and their scaling with rising temperature (Supplementary Fig. 13).

Supplementary Fig. 14 explore the differences between CMIP5 and CMIP6 results. We use the CMIP5 outputs to reproduce the scaling results in Supplementary Fig. 14.

Supplementary Figs. 15-17 serve for the “Discussion” section. We investigate the uncertainty of T_{pp} (Supplementary Fig. 11) and EPS (Supplementary Fig. 12) changes between the 1985-2014 reference and 2071-2100 future periods and find the dynamic term plays a dominant role. We also analyze future changes in precipitation and runoff (Supplementary Fig. 13) to show the shifting EPS impacts on the water cycle.

Supplementary Tables. 1-2 provide the information of the CMIP6 (Supplementary Table. 1) and CMIP5 (Supplementary Table. 2) ensembles used in the study.

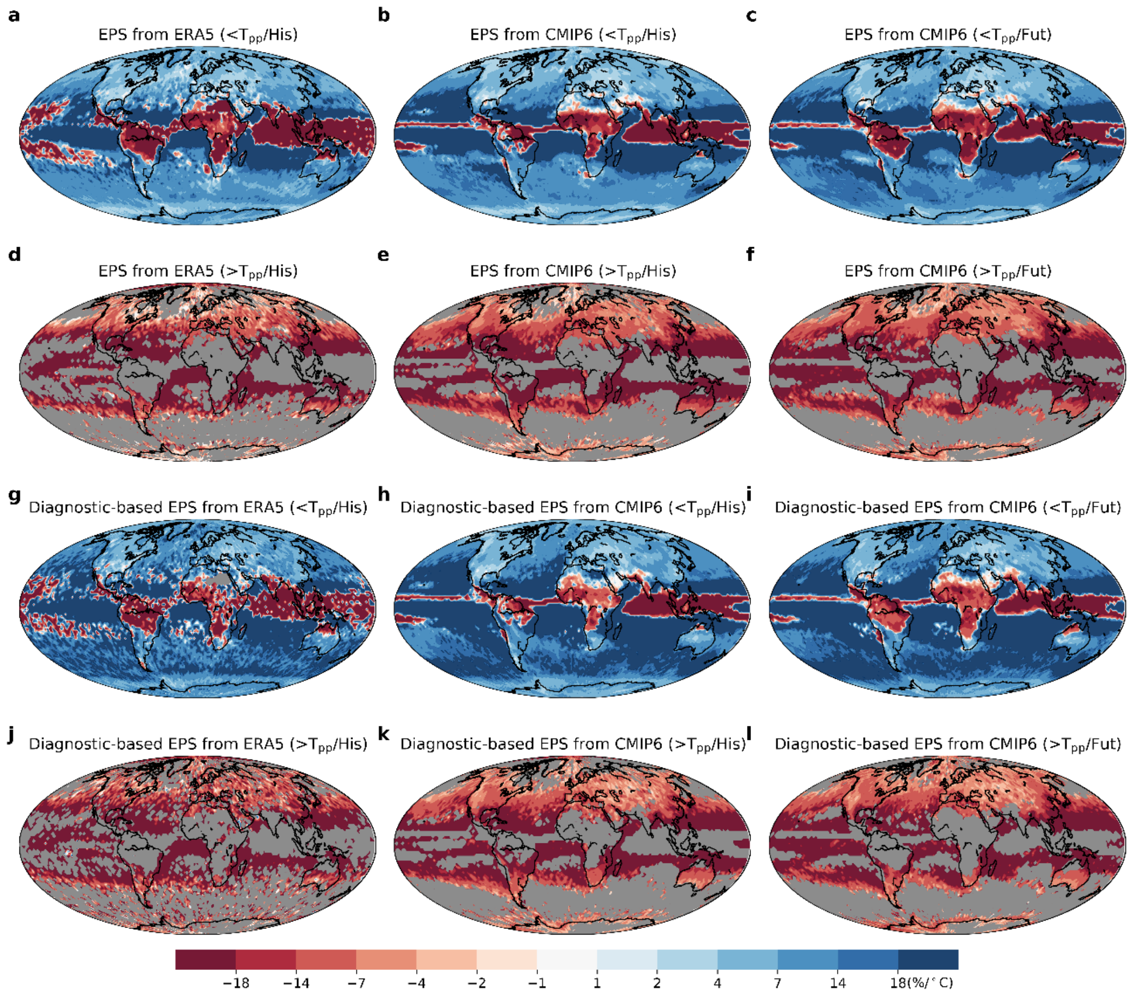

Supplementary Figure 1. Consistency of spatial patterns in actual and diagnostic-based extreme precipitation sensitivity (EPS). a-c 99th-percentile precipitation-temperature scaling rate (EPS) from ERA5 reanalysis (**a**) and CMIP6 multi-model ensemble mean (**b-c**) before T_{pp} ($<T_{pp}$). **d-f** The same as (**a-c**), but after T_{pp} ($>T_{pp}$). **g-i** 99th-percentile precipitation-temperature scaling rate (EPS) based on the physical diagnostic (Diagnostic-based) from ERA5 reanalysis (**g**) and CMIP6 multi-model ensemble mean (**h-i**) before T_{pp} ($<T_{pp}$). **j-l** The same as (**g-i**), but after T_{pp} ($>T_{pp}$).

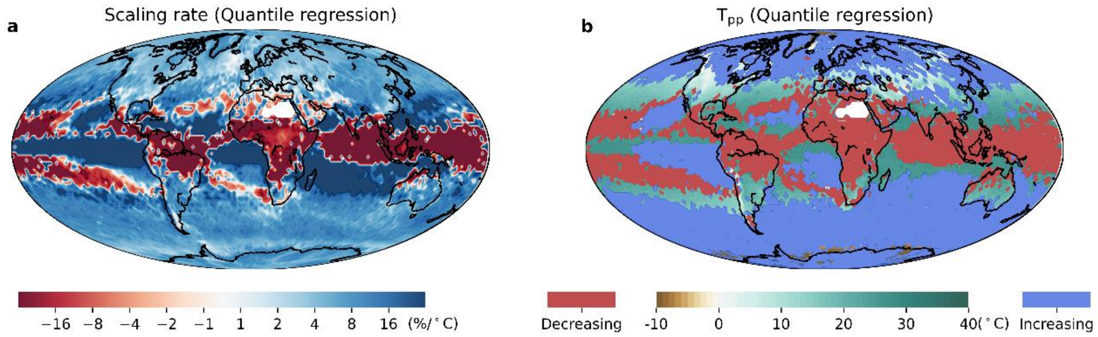

Supplementary Figure 2. Extreme precipitation sensitivity (EPS) and the peak-point temperature (T_{pp}) based on the quantile regression and the Akaike information criterion during the reference 1985-2014 period using ERA5 dataset.

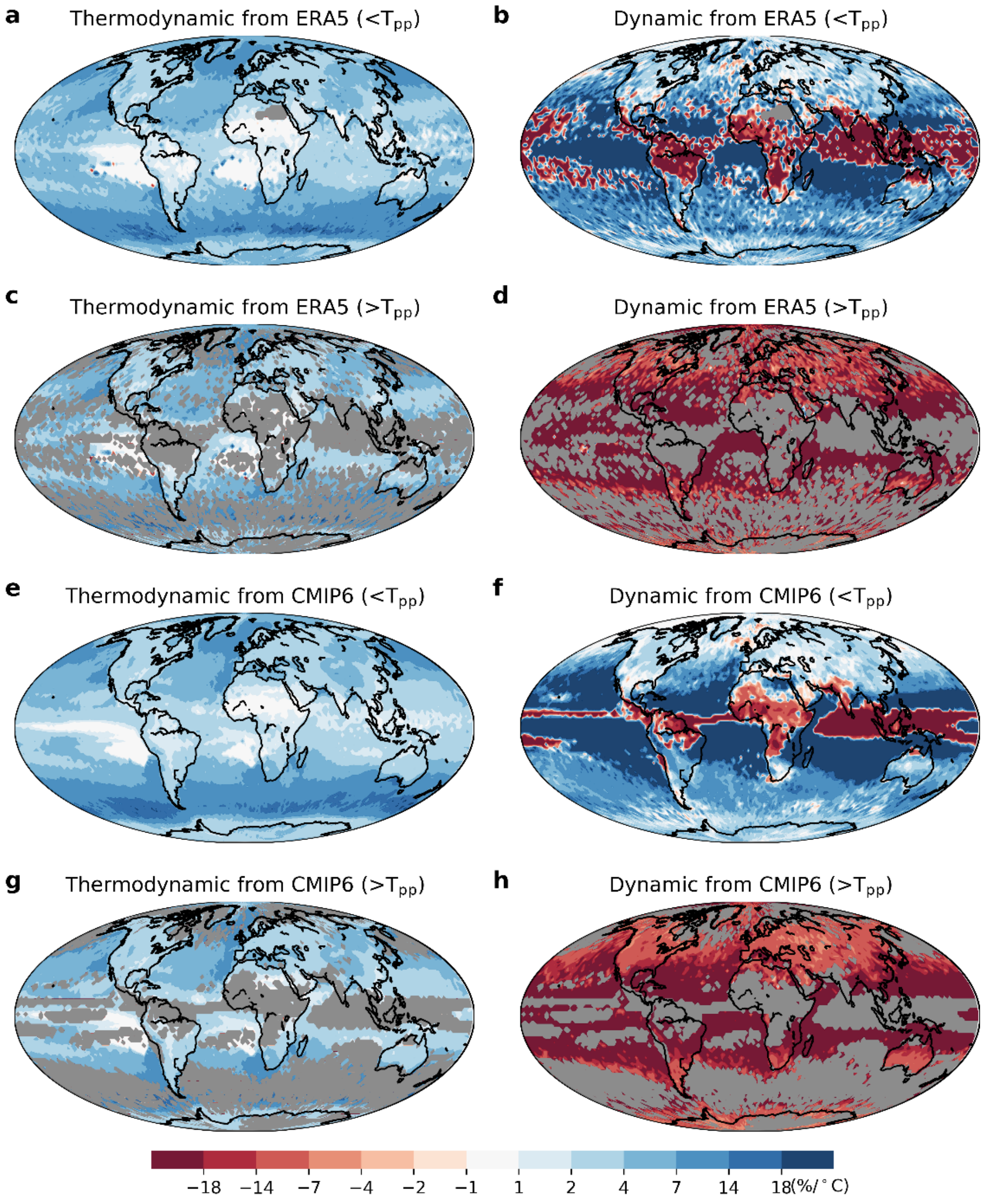

Supplementary Figure 3. Thermodynamic versus dynamic contribution to extreme precipitation sensitivity (EPS) in ERA5 and CMIP6 during the reference 1985-2014 period. **a-b** Thermodynamic versus dynamic contribution to extreme precipitation-temperature scaling rate (EPS) from ERA5 reanalysis before T_{pp} ($<T_{pp}$). **c-d** The same as (**a-c**), but after T_{pp} ($>T_{pp}$). **e-f** Thermodynamic versus dynamic contribution to extreme precipitation-temperature scaling rate (EPS) from CMIP6 dataset before T_{pp} ($<T_{pp}$). **g-h** The same as (**e-f**), but after T_{pp} ($>T_{pp}$). Monotonic scaling types in (**c**,**d**,**g**,**h**) are masked by grey.

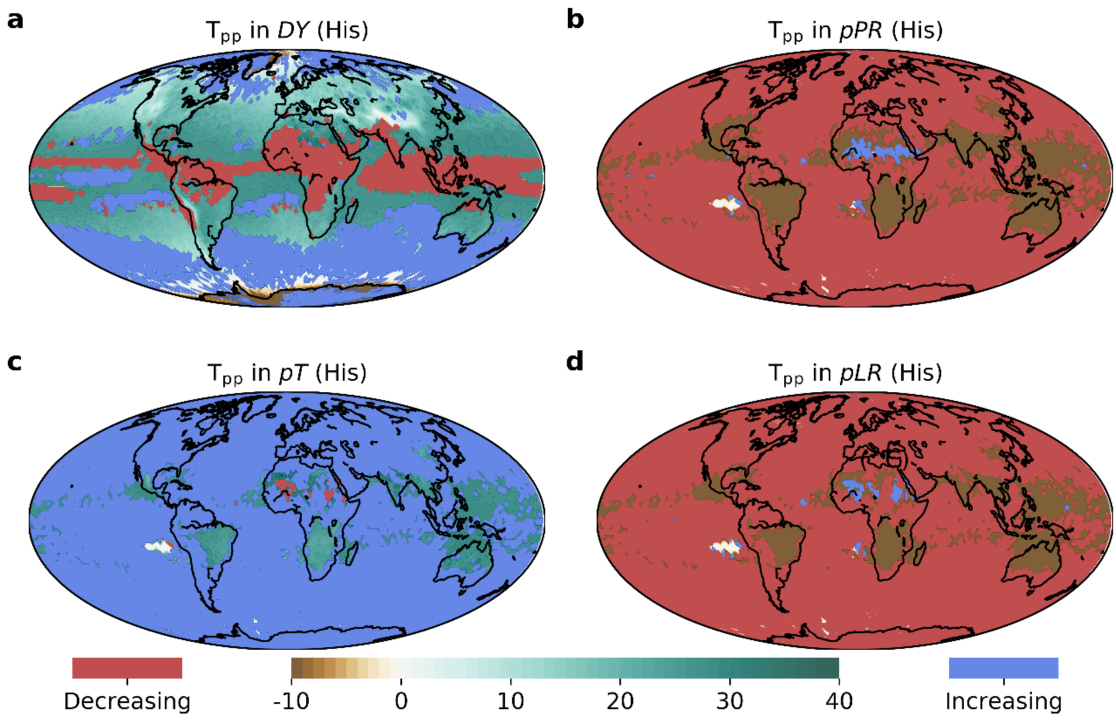

Supplementary Figure 4. The peak-point temperature (T_{pp}) in thermodynamic and dynamic components under 1985-2014 reference climate. **a** The peak point temperature (T_{pp}) in thermodynamic temperature component (*pT*). **b** T_{pp} in lapse rate component (*pLR*). **c** T_{pp} in thermodynamic pressure component (*pPR*). **d** T_{pp} in dynamic component (*DY*). The monotonically decreasing (Decreasing) and increasing (Increasing) EPS regime is masked by the vermillion (blue-violet) color.

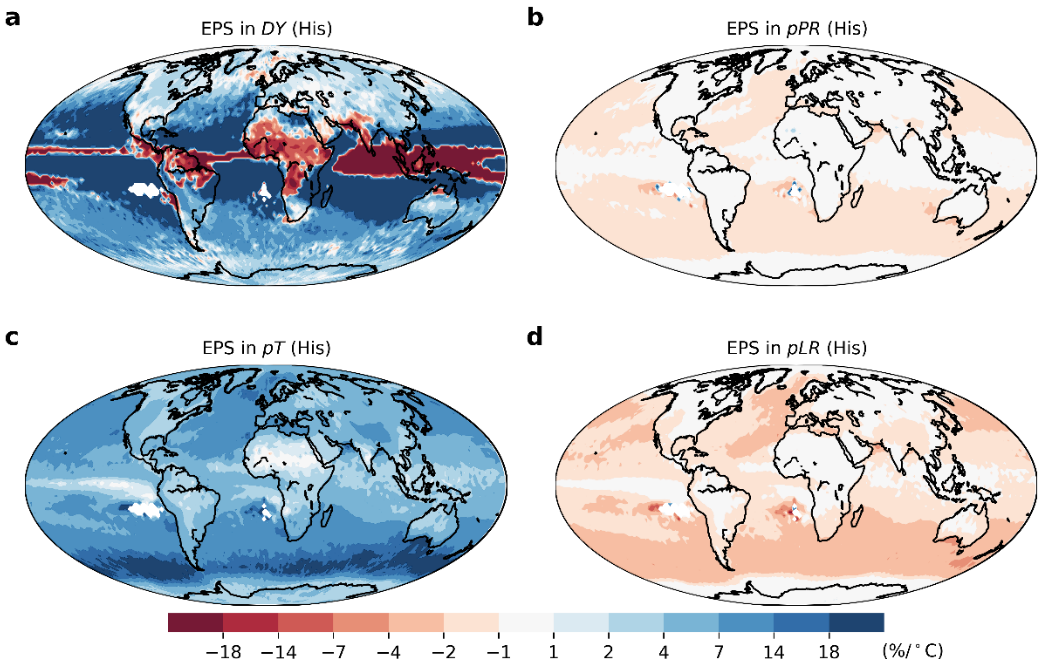

Supplementary Figure 5. Contributions of dynamic versus thermodynamic components to extreme precipitation sensitivity (EPS) under the 1985-2014 reference climate. a Scaling rate of the thermodynamic temperature component (*pT*) contributing to extreme precipitation sensitivity (EPS). **b** The same as (**a**), but for the lapse rate component (*pLR*). **c** The same as (**a**), but for the thermodynamic pressure component (*pPR*). **d** The same as (**a**), but for the dynamic component.

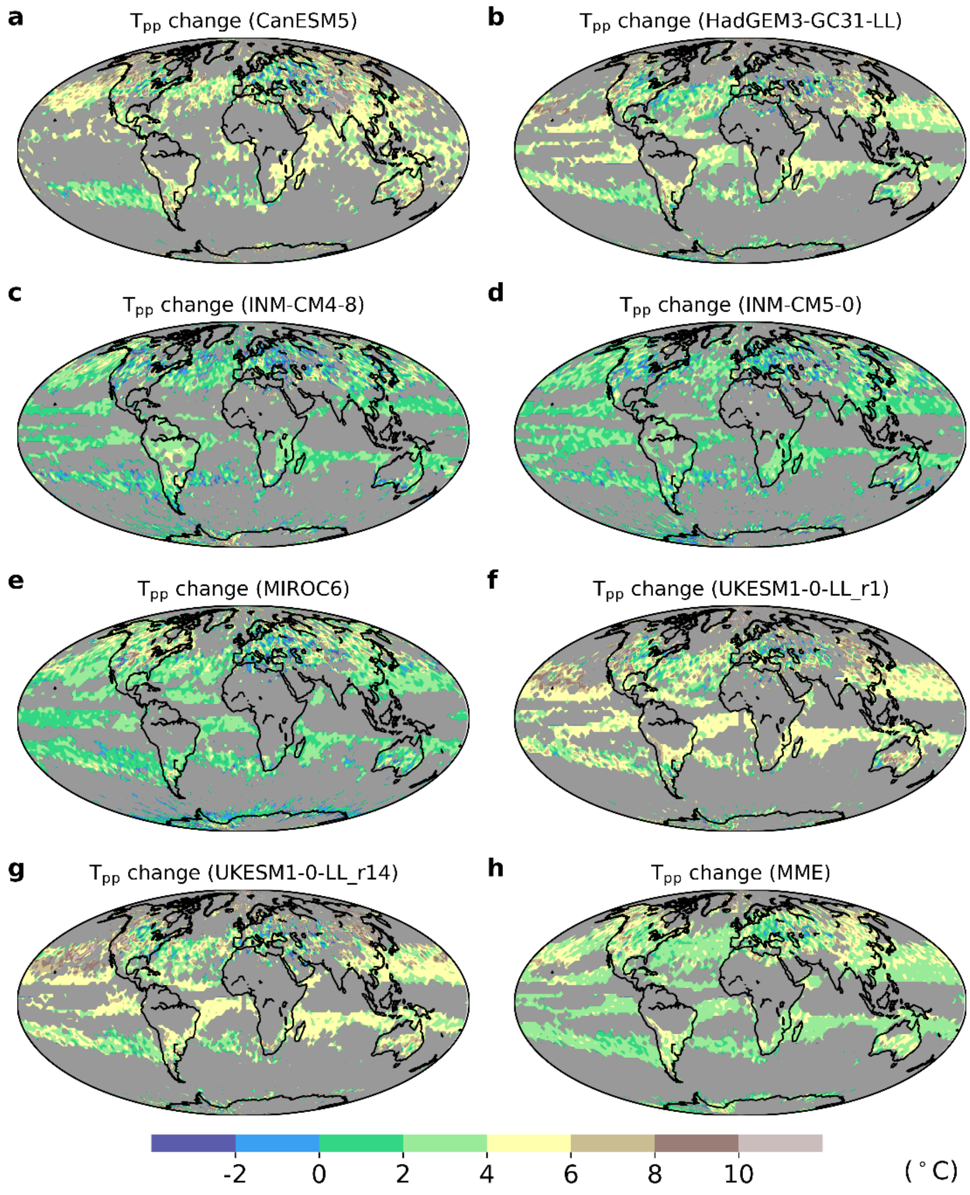

Supplementary Figure 6. The peak-point temperature (T_{pp}) changes between the 1985-2014 reference and the 2071-2100 future periods within CMIP6 dataset. a T_{pp} changes projected by CanESM5 model. **b-g** The same as (**a**), but for HadGEM3-GC31-LL, INM-CM4-8, INM-CM5-0, MIROC6, UKESM1-0-LL (*r1*) and UKESM1-0-LL (*r14*), respectively. Basic model information is shown in Table S1. **h** The same as (**a**), but for multi-model ensemble mean results (MME). The T_{pp} changes are only presented in the hook-like regime spanning both reference and future periods (using the right-side colorbar). Otherwise, in areas characterized by the monotonically increasing and decreasing regimes, the changing behaviors are masked by grey.

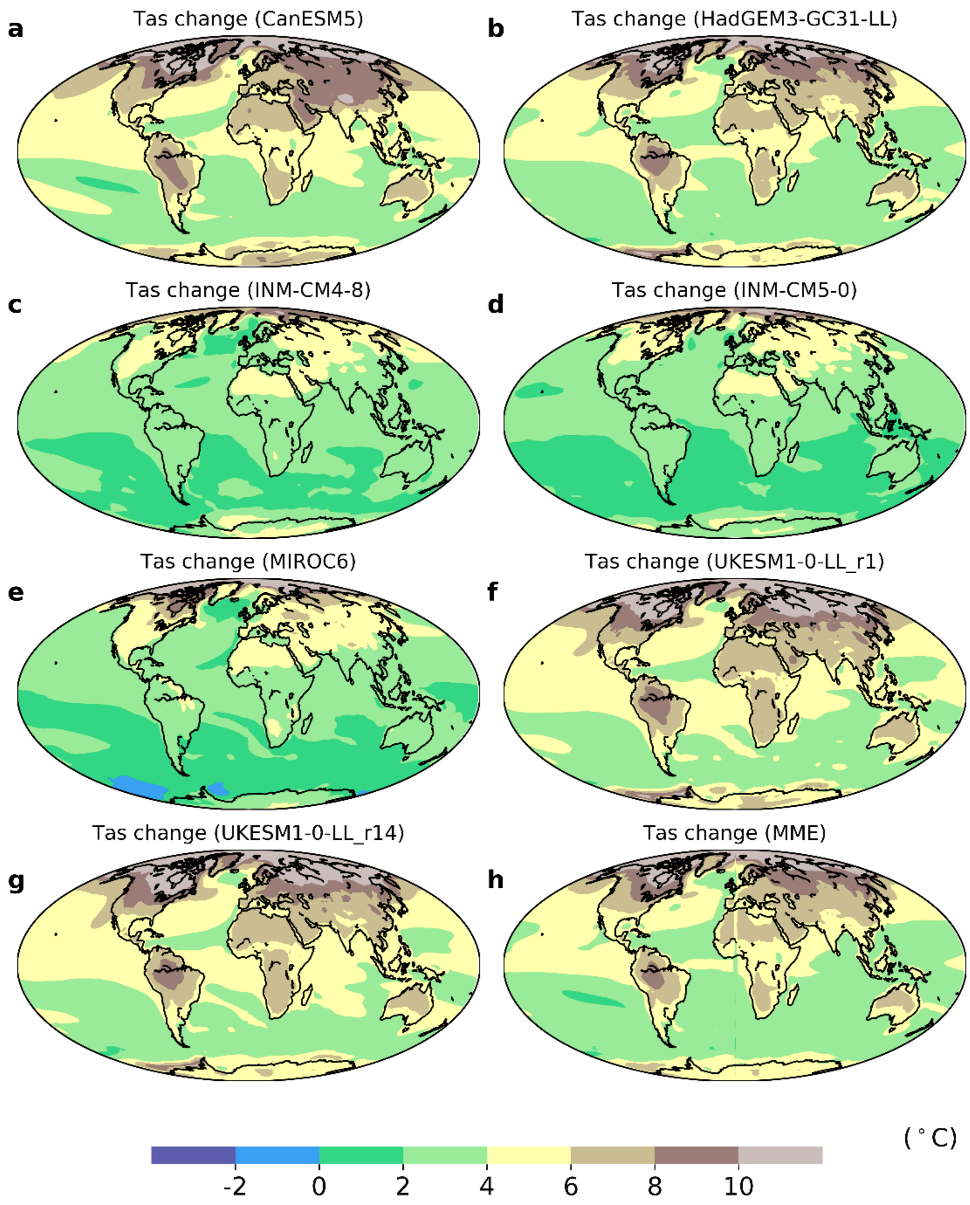

Supplementary Figure 7. The local mean temperature (T_{as}) changes between the 1985-2014 reference and the 2071-2100 future periods within the CMIP6 dataset. a T_{as} changes projected by CanESM5 model. **b-g** The same as (**a**), but by HadGEM3-GC31-LL, INM-CM4-8, INM-CM5-0, MIROC6, UKESM1-0-LL (*r1*) and UKESM1-0-LL (*r14*) respectively. Basic model information is shown in Supplementary Table 1. **h** The same as (**a**), but for multi-model ensemble mean results (MME).

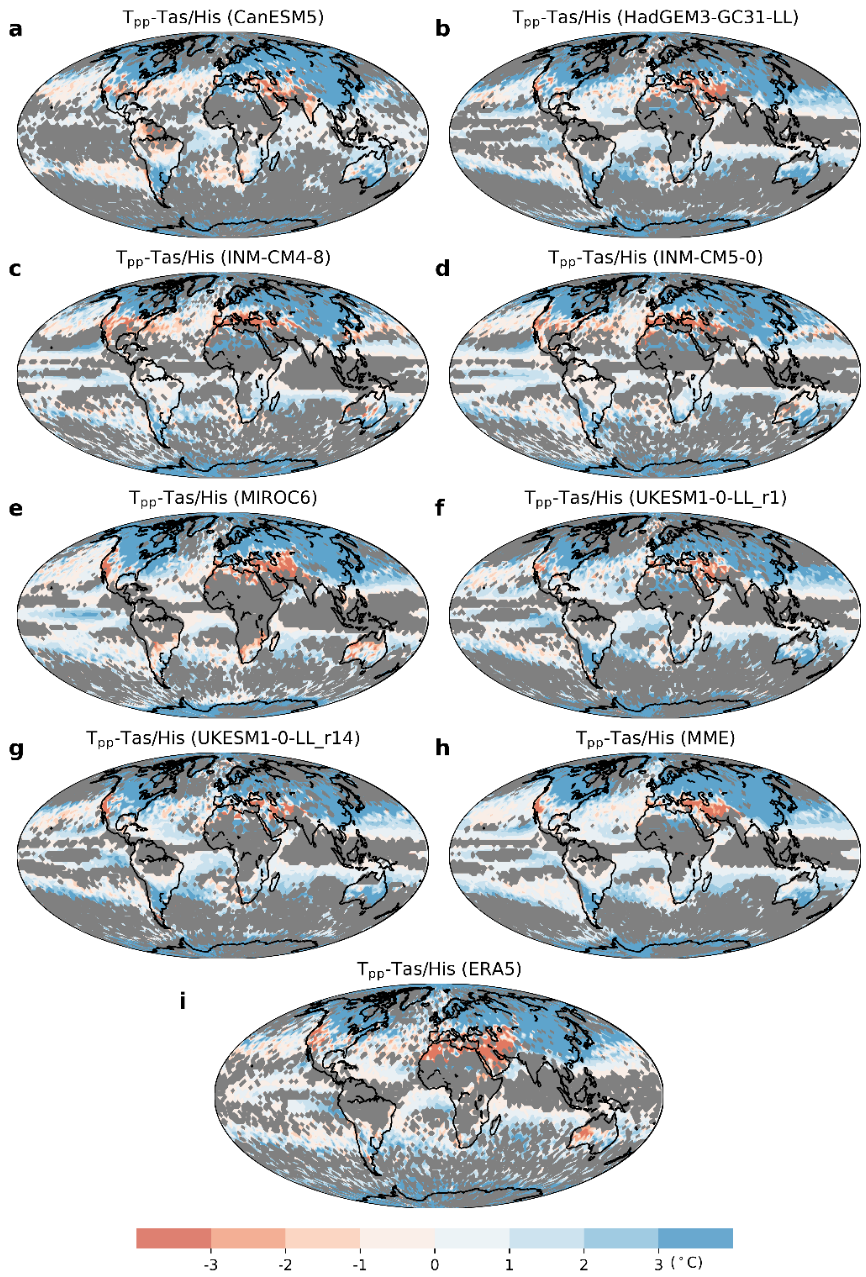

Supplementary Figure 8. Differences between the peak-point temperature (T_{pp}) and the local mean temperature (T_{as}) during the 1985-2014 reference period within the CMIP6 dataset. a Differences (**T_{pp}** minus T_{as}) simulated by the CanESM5 model. **b-g** The same as (**a**), but by HadGEM3-GC31-LL, INM-CM4-8, INM-CM5-0, MIROC6, UKESM1-0-LL (*r1*) and UKESM1-0-LL (*r14*), respectively. Basic model information is shown in Supplementary Table 1. (**h**) The same as (**a**), but for multi-model ensemble mean results (MME). The results are only for regions showing a hook structure spanning both reference and future periods. Otherwise, in areas characterized by the monotonically increasing and decreasing regimes, the changing behaviors are masked by grey.

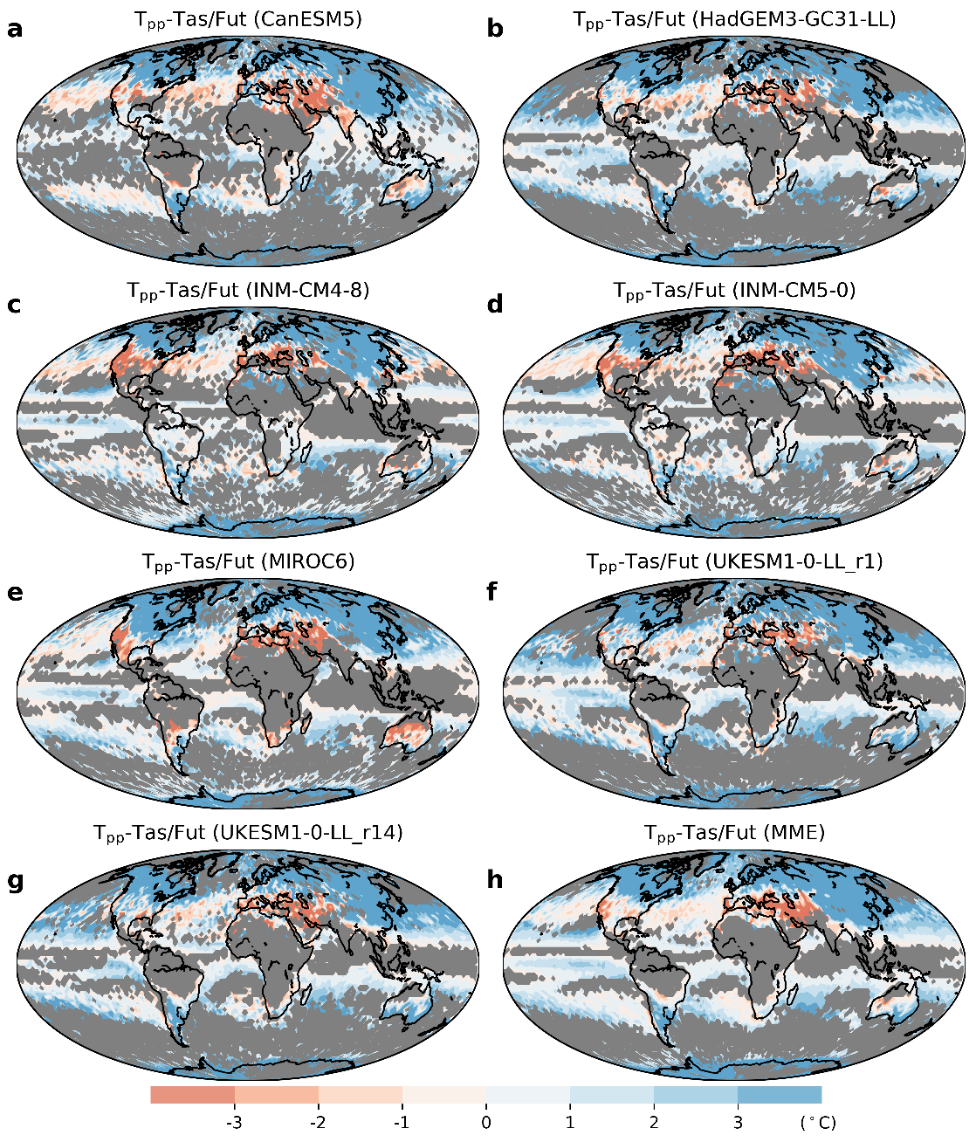

Supplementary Figure 9. Differences between the peak-point temperature (T_{pp}) and the local mean temperature (T_{as}) during the 2071-2100 future period within the CMIP6 dataset. a Differences (T_{pp} minus T_{as}) projected by the CanESM5 model. **b-g** The same as (**a**), but by HadGEM3-GC31-LL, INM-CM4-8, INM-CM5-0, MIROC6, UKESM1-0-LL (*r1*) and UKESM1-0-LL (*r14*), respectively. Basic model information is shown in Supplementary Table 1. **h** The same as (**a**), but for multi-model ensemble mean results (MME). The results are only for regions showing a hook structure spanning both reference and future periods. Otherwise, in areas characterized by the monotonically increasing and decreasing regimes, the changing behaviors are masked by grey.

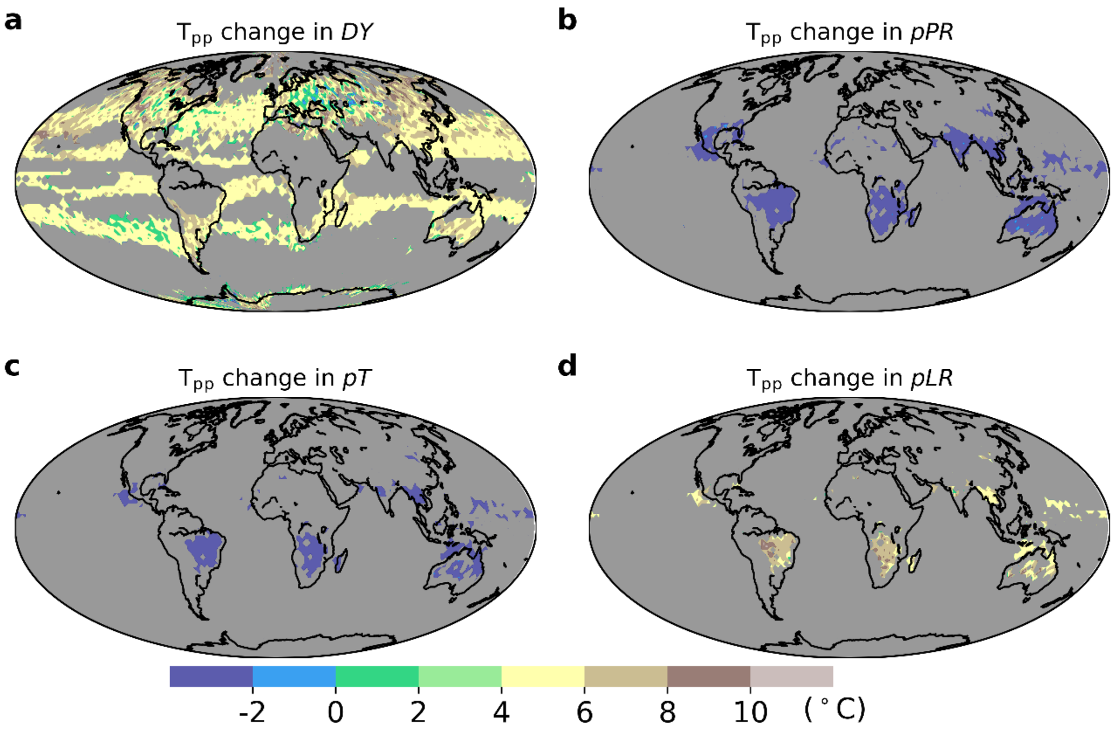

Supplementary Figure 10. The peak-point temperature (T_{pp}) changes in thermodynamic and dynamic components between 1985-2014 reference and 2071-2100 future periods within CMIP6 dataset. a T_{pp} changes in thermodynamic temperature component (*pT*). **b** T_{pp} changes in lapse rate component (*pLR*). **c** T_{pp} changes in thermodynamic pressure component (*pPR*). **d** T_{pp} changes in dynamic component (*DY*). The T_{pp} changes are only presented in the hook-like regime spanning both reference and future periods. Otherwise, in areas characterized by the monotonically increasing and decreasing regimes, the changing behaviors are masked by grey.

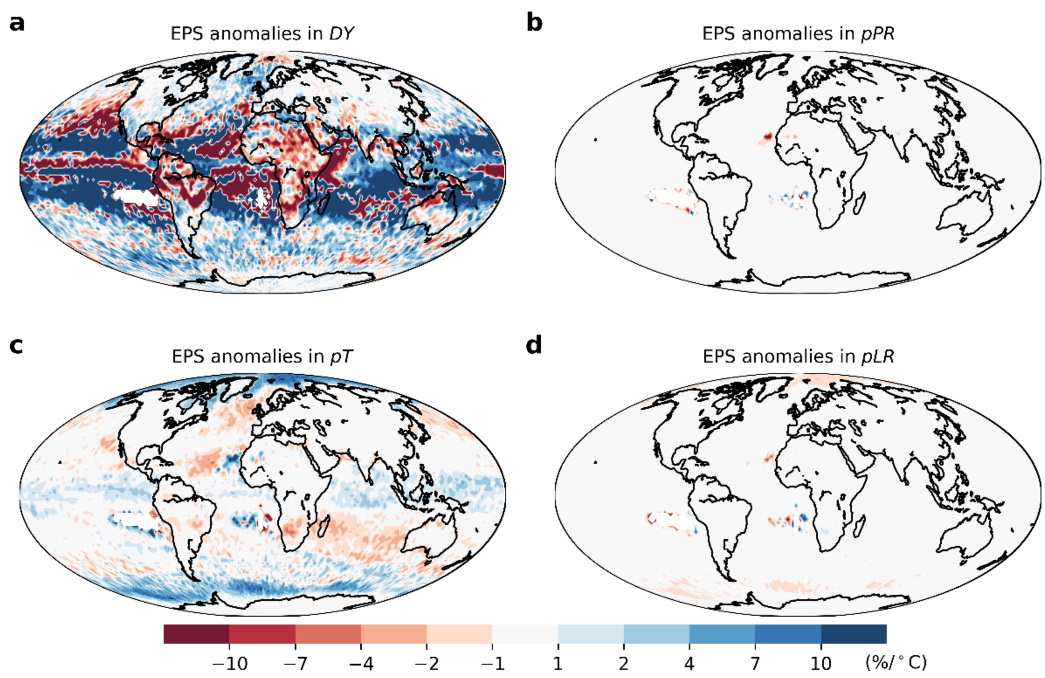

Supplementary Figure 11. Contributions of dynamic versus thermodynamic components to extreme precipitation sensitivity (EPS) anomalies between 1985-2014 reference and 2071-2100 future periods within CMIP6 dataset. a Scaling rate anomalies (i.e., subtracting reference period value) of the thermodynamic temperature component (*pT*) contributing to EPS anomalies. **b** The same as (**a**), but for the lapse rate component (*pLR*). **c** The same as (**a**), but for the thermodynamic pressure component (*pPR*). **d** The same as (**a**), but for the dynamic component.

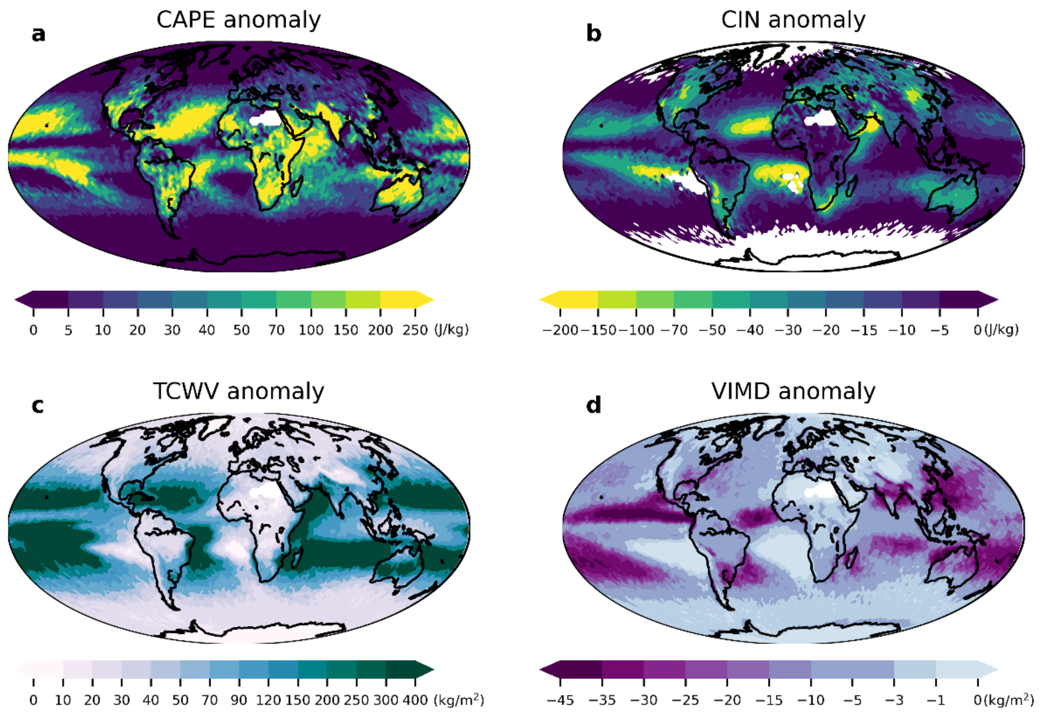

Supplementary Figure 12. The thermodynamic and dynamic environment corresponding to extreme precipitation during the reference 1985-2014 period in the ERA5 dataset. a, Convective available potential energy (CAPE) anomalies corresponding to extreme precipitation. Here anomalies denote CAPE values during extreme precipitation minus temporal mean values during the reference period. **b**, Convective inhibition (CIN) anomalies corresponding to extreme precipitation. **c**, Total column water vapor (TCWV) anomalies corresponding to extreme precipitation. **d**, Vertically integrated moisture divergence (VIMD) anomalies corresponding to extreme precipitation. Negative values denote moisture convergence.

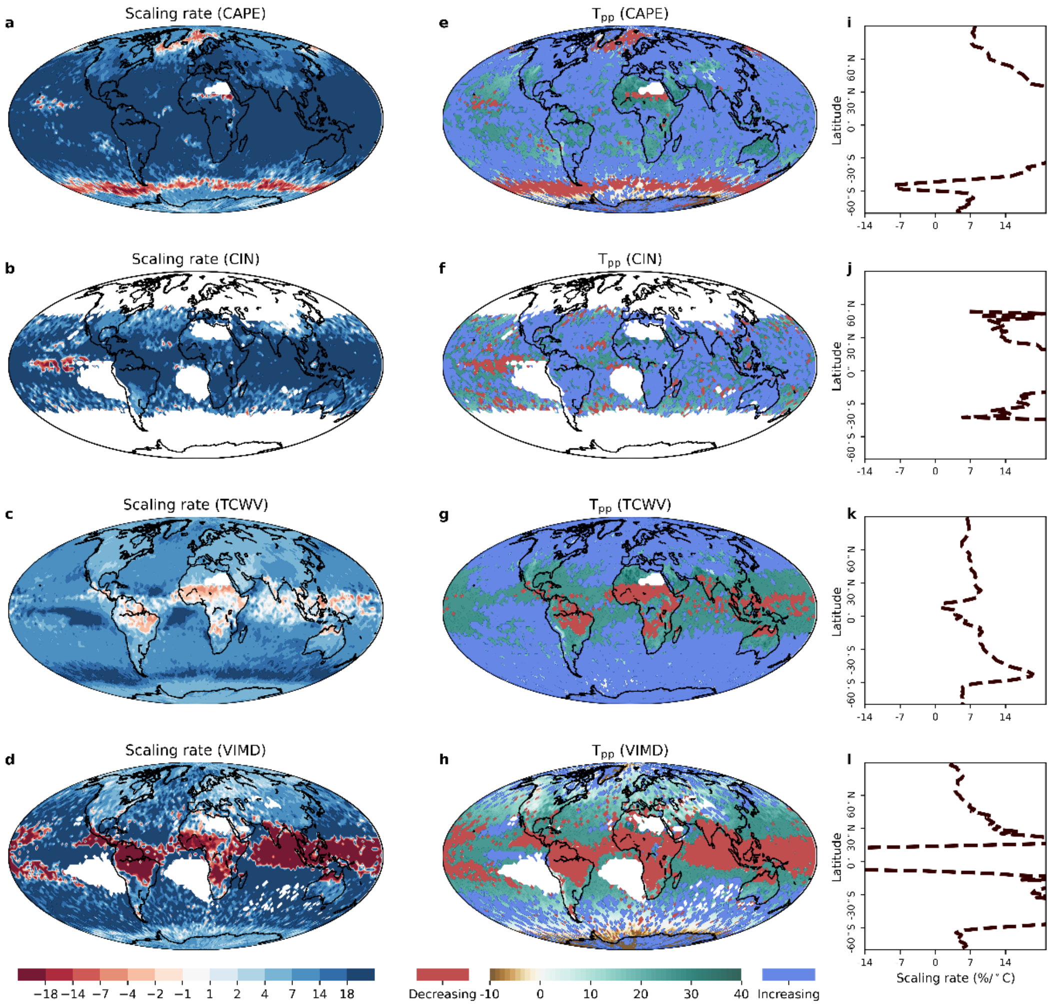

Supplementary Figure 13. The thermodynamic and dynamic variables scaling with rising temperature during 1985-2014 from the ERA5 dataset. a-d, the scaling rate of CAPE, CIN, TCWV and VIMD with rising temperature. **e-h,** the T_{pp} of CAPE, CIN, TCWV and VIMD. i-l, the zonal median scaling rate of CAPE, CIN, TCWV and VIMD. Here, all these variables are corresponding to 99th percentile daily precipitation.

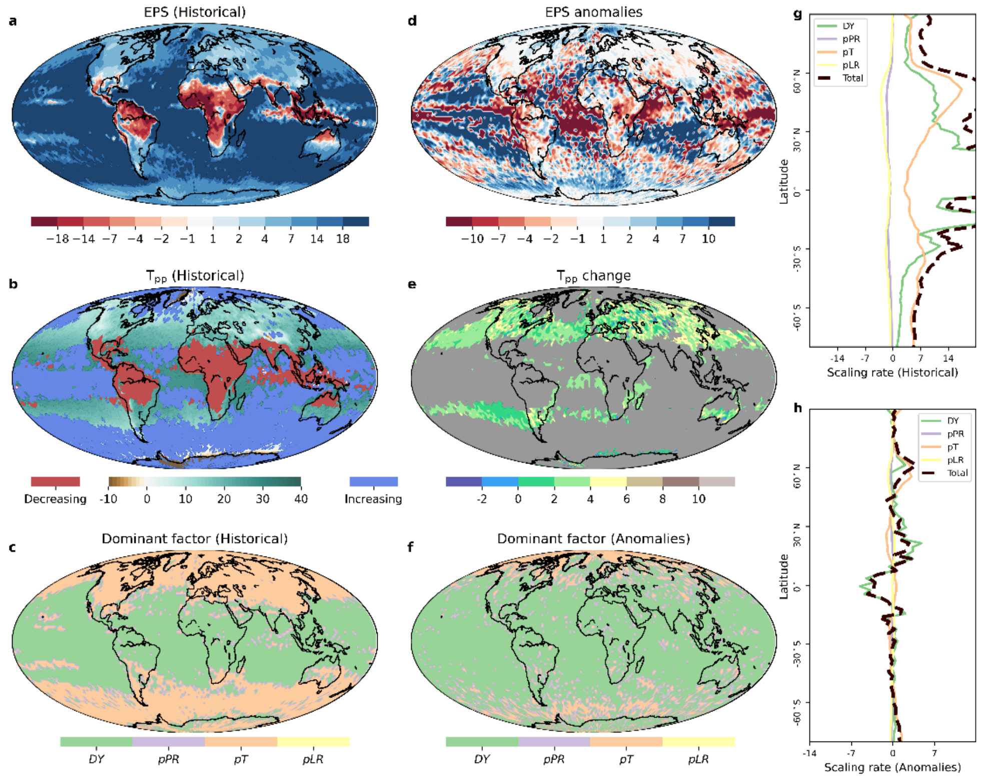

Supplementary Figure 14. Extreme precipitation sensitivity (EPS), the peak-point temperature (T_{pp}) and dominant factor during the reference 1985-2014 period (a-c) and associated changes during the future 2071-2100 future period relative reference period (d-f). **g**, Zonal EPS and the *DY*, *pPR*, *pT* and *pLR* contributions to zonal EPS during the reference period. **h**, Zonal EPS anomalies and the associated *DY*, *pPR*, *pT* and *pLR* contributions to zonal EPS anomalies during the future 2071-2100 future period relative reference period.

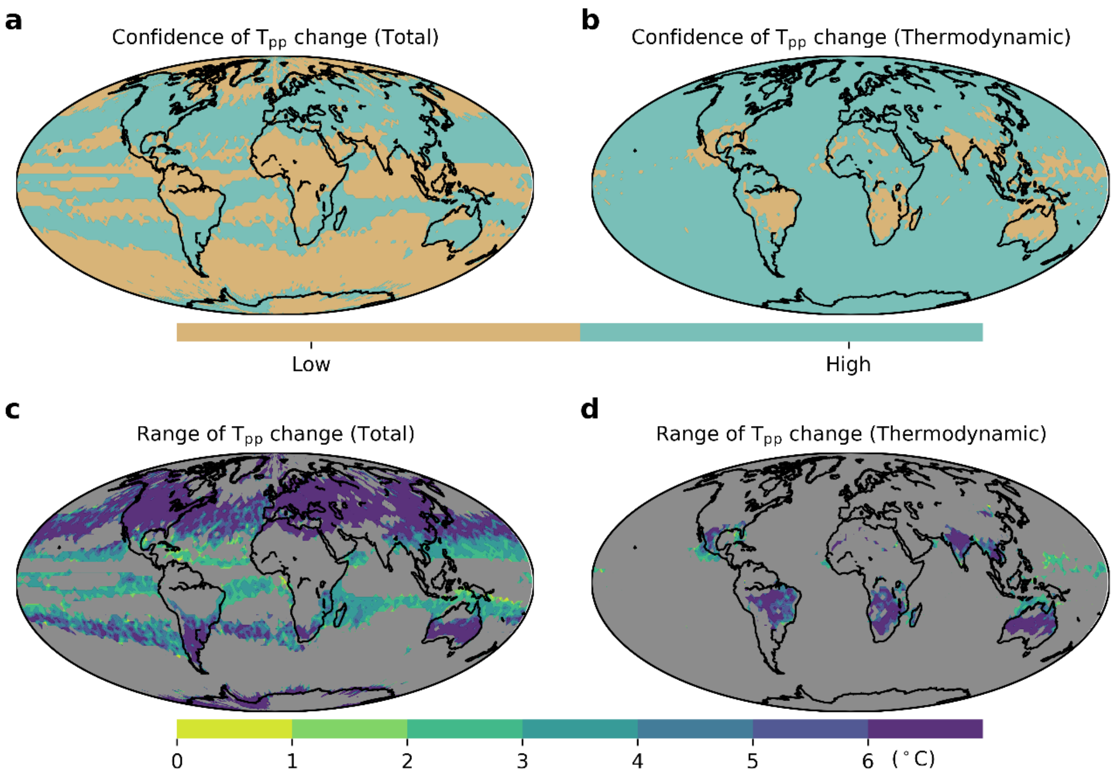

Supplementary Figure 15. Uncertainty of the peak-point temperature (T_{pp}) changes forcing by total and thermodynamic conditions. a-b Confidence in the sign of T_{pp} changes for total and thermodynamic conditions (if at least 3 of 5 models exhibiting the same sign, confidence is deemed as high). **c-d** Range of T_{pp} changes (maximum minus minimum values in T_{pp} changes projected by the CMIP6 multi-model ensemble). The monotonically increasing and decreasing scaling regimes are masked by grey in **c-d**.

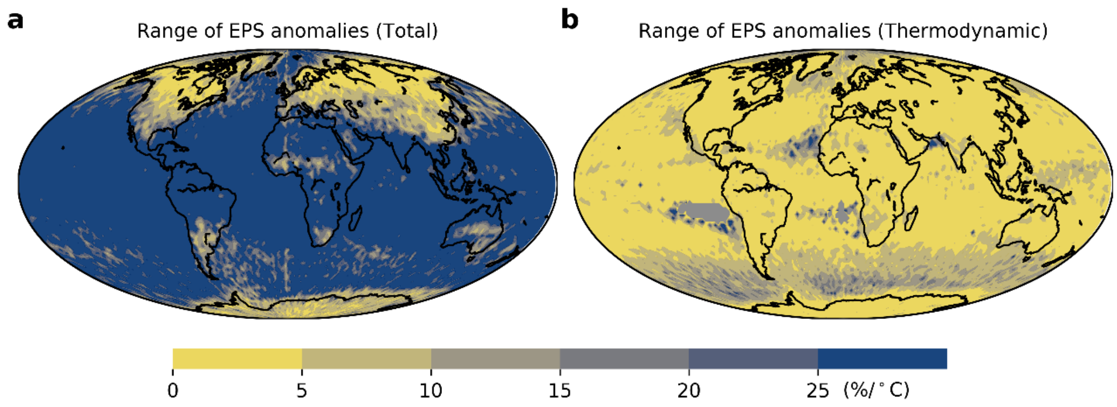

Supplementary Figure 16. Uncertainty of extreme precipitation sensitivity (EPS) anomalies forcing by total and thermodynamic conditions. a Range of EPS anomalies forcing by total conditions (maximum minus minimum values in EPS anomalies projected by the CMIP6 multi-model ensemble). **b** The same as (**a**), but for thermodynamic conditions.

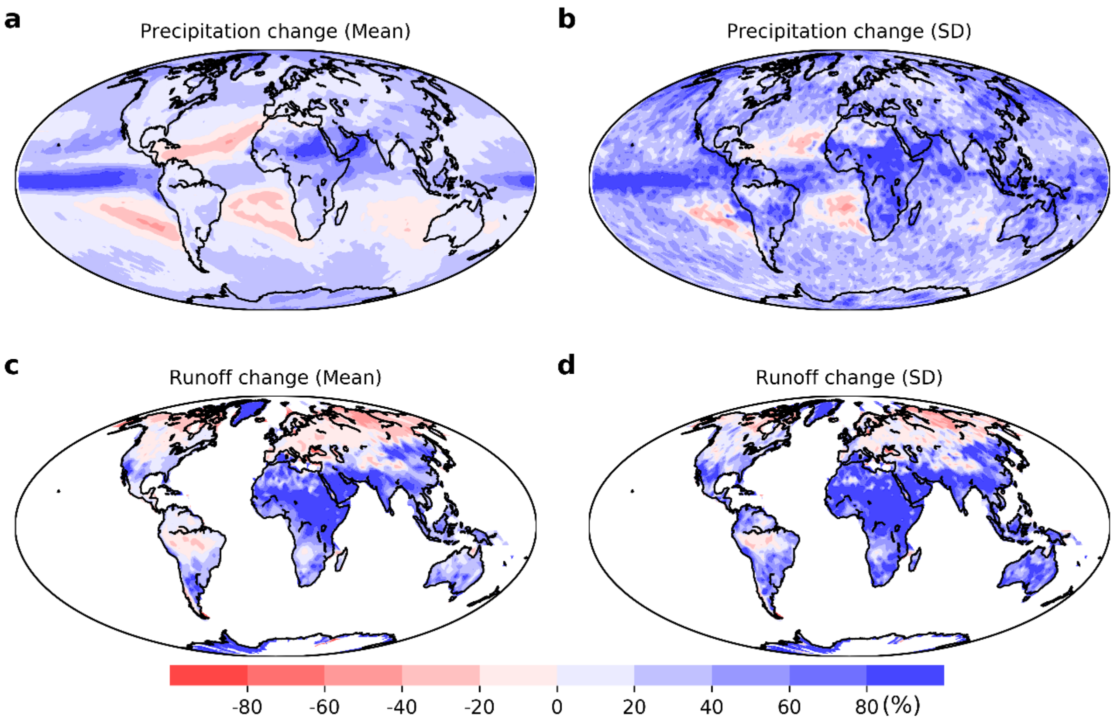

Supplementary Figure 17. Extreme precipitation and runoff changes between reference (1985-2014) and future (2071-2100) periods within the CMIP6 dataset. a-b The 99th -percentile extreme precipitation changes for mean (**a**) and standard deviation (**b**) values from the multi-model ensemble mean. **c-d** The same as (**a-b**), but the 99th-percentile extreme runoff changes.

Supplementary Table 1. **Basic information about the available CMIP6 model outputs**

NO	Institution	Model name	Ensemble	Resolution
				Lon.×Lat.×Plev.
1	Canadian Centre for Climate Modelling and Analysis	CanESM5	*r1i1p2f1*	128×90×19
2	Institute for Numerical Mathematics, Russian Academy of Science	INM-CM4-8	*r1i1p1f1*	180×120×19
3		INM-CM5-0	*r1i1p1f1*	
4	Japan agency for marine-earth science and technology	MIROC6	*r1i1p1f1*	256×128×19
5	Met Office Hadley Centre	HadGEM3-GC31-LL	*r1i1p1f3*	192×144×19
6		UKESM1-0-LL	*r1i1p1f2*	
7			*r14i1p1f2*	

Supplementary Table 2. **Basic information about the available CMIP5 model outputs**

NO	Institution	Model name	Ensemble	Resolution
				Lon.×Lat.×Plev.
1	Canadian Centre for Climate Modelling and Analysis	CanESM2	*r1i1p1f1*	128×64×8
2	Centro Euro-Mediterraneo sui Cambiamenti Climatici	CMCC-CESM	*r1i1p1f1*	96×48×11
3	NOAA Geophysical Fluid Dynamics Laboratory	GFDL-CM3	*r1i1p1f1*	144×90×8
4	Japan agency for marine-earth science and technology	MIROC5	*r1i1p1f1*	180×90×8
5	Institut Pierre Simon Laplace	IPSL-CM5A-MR	*r1i1p1f1*	180×90×8
6	Max Planck Institute for Meteorology	MPI-ESM-MR	*r1i1p1f1*	192×96×15
